# The role of NEDD4-mediated ubiquitination of AQP5 in allergic rhinitis: implications for nasal mucosal barrier function

**DOI:** 10.3389/fimmu.2026.1908261

**Published:** 2026-09-17

**Authors:** Jinchao Wang, Tiesuo Chen, Fang He, Xiaoyun Feng

**Affiliations:** 1 Audiology and Hearing Care Center, Huizhou Central People’s Hospital, Huizhou, China; 2 Department of Surgery, Gongzhuang Town Health Center, Boluo County, Huizhou, China; 3 Department of Anesthesia, Huizhou Central People’s Hospital, Huizhou, China; 4 Department of Pathology, ZhongShan-XuHui Hospital, FuDan University, Shanghai, China

**Keywords:** allergic rhinitis, AQP5, nasal mucosal barrier, NEDD4, p38 MAPK signaling

## Abstract

**Background:**

Allergic rhinitis (AR) pathology is mainly due to disruption of the nasal epithelial barrier, but the role of aquaporin 5 (AQP5) is not fully understood. We propose that the E3 ubiquitin ligase NEDD4 is responsible for the degradation of AQP5 and thus affects barrier homeostasis in AR.

**Methods:**

Human nasal epithelial cells and ovalbumin-induced Sprague-Dawley rats were used as *in vitro* and *in vivo* models, respectively. Control and AR groups were compared with siNEDD4 and ovAQP5 groups using co-immunoprecipitation, confocal microscopy, and ubiquitination assays to assess molecular interactions. Transepithelial electrical resistance (TER), MUC5AC secretion, and cytokine release were used to measure barrier function.

**Results:**

We demonstrated that NEDD4 directly interacts with and ubiquitinates AQP5 at lysine 257, leading to its proteasomal degradation. NEDD4 was upregulated by IL-13, and this upregulation did not alter AQP5 mRNA levels. Decreasing NEDD4 or increasing AQP5 expression restored barrier integrity and inhibited MUC5AC and IL-6/IL-8 synthesis and concomitantly suppressed p38 MAPK activation; this suppression is consistent with AQP5-mediated signaling. Inhibiting p38 with SB203580 restored barrier function and counteracted mucus hypersecretion. Nasal administration of siNEDD4 or ovAQP5 in rats relieved allergic reactions, reduced inflammatory cell infiltration, and normalized NEDD4/p-p38/AQP5 expression.

**Conclusions:**

We have identified the NEDD4-AQP5 axis as a crucial driver of AR and a potential therapeutic target.

## 1 Introduction

Allergic rhinitis (AR) is a chronic inflammatory disease of the nose that occurs due to a Type I hypersensitivity reaction and is mainly caused by immunoglobulin E (IgE). The primary sign of allergic rhinitis is a loss of the barrier function of the nasal mucous membrane and excessive activity of the Th2 immune response leading to changes in inflammatory mediator secretion (1, 2). As the first line of defense against external allergens, the structure and function of this barrier must be stable so that the barrier cannot be penetrated by allergens and immune tolerance can be maintained (3). Whether the mucosa can secrete mucus smoothly, whether the tight junctions are intact, and whether there is transport of water are all related to the achievement of homeostasis, but the molecular mechanism for this regulation has not been fully explored.

Aquaporins (AQPs) are transmembrane proteins that conduct water and regulate the balance of body fluids; thus, they have recently been studied (4). Aquaporin 5 (AQP5) is relatively abundant in the nasal epithelium and regulates both the movement of water and mucus secretion. It is crucial for maintaining the integrity of the mucosal barrier (5, 6). Clinically, it has been found that AQP5 expression in the nasal mucosa of AR patients is reduced, and this decrease is associated with increased epithelial permeability and more severe symptoms (7). The mechanism by which AR causes the downregulation of AQP5 is not well understood, and the role of ubiquitination has not been investigated.

Besides transcription alterations, there are other post-translational modifications (PTMs) such as phosphorylation, ubiquitination, small ubiquitin-like modifier modifications (SUMOylation), and acetylation, all of which need to be dynamically carried out to regulate the function, subcellular location, stability, etc., of epithelial barrier proteins (8–10). For instance, SUMOylation alters the membrane localization of β-catenin in airway epithelial cells, thereby modulating Wnt signaling (8); ubiquitination targets claudin-1 for degradation, compromising tight junction integrity (9); and histone deacetylase (HDAC)-mediated deacetylation epigenetically governs the expression of tight junction molecules, with markedly elevated activity observed in AR (10). These examples illustrate that distinct PTM types cooperatively fine-tune the epithelial barrier, yet whether AQP5 undergoes specific PTMs in AR and the regulatory mechanisms remain unexplored.

One major type of post-translational modification is ubiquitination; it can promote the breakdown of some inflammatory proteins (11). Neural precursor cell-expressed developmentally down-regulated 4 (NEDD4) is a HECT-domain E3-ubiquitin ligase that controls epithelial ion and water transport (12). NEDD4 is related to the ubiquitination and degradation of renal aquaporin 2 (AQP2) for the regulation of water reabsorption (13, 14) and also targets p38α to modulate inflammatory signaling (15). Given the structural similarities between AQP2 and AQP5 and the recognized ability of NEDD4 to regulate epithelial aquaporins (16), we hypothesized that NEDD4 serves as an E3 ubiquitin ligase for AQP5 in AR, targeting it for degradation through the ubiquitin-proteasome pathway. Moreover, the elevated levels of Th2 cytokines within the inflamed nasal mucosa prompt us to postulate their potential implications.

This study aims to elucidate how the NEDD4-AQP5 axis drives the progression of AR. We propose that the inflammatory condition in AR leads to an increase in NEDD4, which in turn alters AQP5 through a ubiquitin-dependent pathway. Through a combination of *in vitro* and *in vivo* experiments, it was discovered that this degradation process interferes with water transport and barrier function in the epithelium; as a result, mucosal inflammation increases due to activation of the p38 mitogen-activated protein kinase (p38 MAPK) signaling pathway. Based on the analysis of the results, there is damage to the epithelial barrier of the nasal cavity, and Th2 cytokines play a central role in this. We need to identify the molecular targets of the disease for its treatment. In the medical literature, it has been found that AQP5 expression in the nasal mucosa of AR patients is altered, but the specific regulatory mechanism has not been identified. This study addresses this problem by showing that NEDD4 acts as the E3 ubiquitin ligase for AQP5 in AR.

## 2 Materials and methods

### 2.1 Cell culture and treatments

Human nasal epithelial cells (HNEpCs) were maintained in DMEM/F12 medium (Gibco, Cat. No. 21331020) supplemented with 10% fetal bovine serum (Gibco, Cat. No. A5256901) and 1% penicillin-streptomycin (HyClone, Cat. No. SV30010), and incubated at 37 °C in a humidified atmosphere with 5% CO_2_.

An *in vitro* AR model was generated by sensitizing HNEpCs with Dermatophagoides pteronyssinus house dust mite (HDM) extract (Cat. No. B82; Greer Laboratories, Lenoir, NC, USA, a subsidiary of Stallergenes Greer), as previously described (17). Lyophilized HDM extract was reconstituted in sterile phosphate-buffered saline (PBS). The stock concentration was adjusted to 10 mg/mL based on total protein content. After sterilization by passage through a 0.22 μm filter, the stock solution was aliquoted for single use (to avoid repeated freeze-thaw cycles) and stored at −80 °C until further use. Upon reaching 70-80% confluence, the cells were stimulated with HDM extract diluted in complete culture medium to a working concentration of 100 μg/mL. The exposure lasted 24 h to trigger epithelial barrier disruption and inflammatory activation. This sensitization protocol was adapted from a previously validated *in vitro* model of HDM-induced nasal epithelial injury (17), with minor modifications. Specifically, the final HDM concentration was set at 100 μg/mL. This dose elicited robust and reproducible barrier dysfunction in HNEpCs, evidenced by reduced transepithelial electrical resistance and enhanced mucus secretion. Inflammatory cytokine release was also induced. Overt cytotoxicity was avoided.

All subsequent experimental manipulations-gene silencing, plasmid overexpression, and cytokine stimulation-were performed as detailed in the following subsections.

### 2.2 Plasmid construction, siRNA, and transfection

The full-length coding sequences of human NEDD4 (for *in vitro* studies) and human AQP5 were amplified by PCR and cloned into the pcDNA3.1-FLAG (Youbio, VT1001) and pcDNA3.1-Myc (Youbio, VT1010) vectors, respectively, generating pcDNA3.1-FLAG-hNEDD4 and pcDNA3.1-Myc-hAQP5 (wild-type). For *in vivo* experiments in rats, rat AQP5 was inserted into the pcDNA3.1-Myc vector to produce pcDNA3.1-Myc-rAQP5. To identify the critical ubiquitination site(s) on AQP5, lysine-to-arginine point mutants of human AQP5 (K240R, K257R, and K258R) were generated using a site-directed mutagenesis kit (Beyotime, D0206S) with the pcDNA3.1-Myc-hAQP5 construct as the template. An HA-tagged ubiquitin plasmid (Youbio, G103538) was used for ubiquitination assays. All constructed plasmids were verified by DNA sequencing. Annotated plasmid maps are provided in **Supplementary Figure S1**.

NEDD4-specific siRNA oligonucleotides (sense: 5′-GGAGAAUUAUGGGUGUCAA-3′, antisense: 5′-UUGACACCCAUAAUUCUCC-3′) were designed and synthesized by Ribobio (Shanghai, China). The chosen sequence showed no significant homology to non-target genes in the BLAST analysis.

For transfection, HNEpCs were seeded in 6-well plates at 2 × 10^5^ cells/well and cultured until 70-80% confluence. For overexpression studies, 2 μg of plasmid DNA per well was used, yielding a final concentration of 1 μg/mL in the culture medium. For gene silencing, NEDD4-specific siRNA was used at a final concentration of 50 nM. In each case, the nucleic acid was diluted in Opti-MEM (Gibco, 31985070) and complexed with Lipofectamine 2000 reagent (Invitrogen, 11668019) according to the manufacturer’s protocol. The transfection mixture was incubated at room temperature for 20 minutes and then added dropwise to the cells. After 4–6 hours, the transfection medium was replaced with fresh complete culture medium. Cells were then cultured for an additional 24–48 hours prior to downstream analyses, unless otherwise specified.

### 2.3 Co-immunoprecipitation (Co-IP) and mass spectrometry

Over the course of 30 minutes, the transfected HNEpCs were lysed on ice in NP-40 lysis buffer (50 mM Tris-HCl, pH 7.4, 150 mM NaCl, 1% Nonidet P-40; Beyotime, P0013F) supplemented with protease (Beyotime, P1005; 100× stock, added at 1:100 dilution, i.e., 10 μL per 1 mL of lysis buffer) and phosphatase inhibitor cocktail (Beyotime, P1081; 100× stock, added at 1:100 dilution, i.e., 10 μL per 1 mL of lysis buffer). The lysate was centrifuged at 13,000 × g for 15 minutes at 4 °C. For each Co-IP, 500 µg of total protein was incubated with 2 µg of specific antibody (anti-FLAG, Sigma-Aldrich, F7425, rabbit polyclonal, ~0.8 mg/mL stock; 2 µg corresponds to ~2.5 µL per reaction; or anti-Myc, Abcam, ab9106; 2 µg per reaction) overnight at 4 °C with rotation, and then 30 µL of Protein A/G magnetic beads (Thermo Scientific™ Pierce, 88802) were added and incubated for 2 hours. The tube was then placed on a magnetic separation rack for 1–2 minutes to pull the beads to the side of the tube. The supernatant was carefully removed, and the beads were washed three times with NETN buffer (50 mM Tris-HCl, pH 8.0, 150 mM NaCl, 1 mM EDTA, 0.5% NP-40) (18), with magnetic separation applied between each wash. The bound proteins were then eluted by boiling in 5× SDS loading buffer.

For mass spectrometry analysis, the Co-IP eluate was separated by SDS-PAGE and stained with Coomassie Brilliant Blue; the band corresponding to the target protein was excised, and the protein was identified by mass spectrometry after trypsin digestion.

### 2.4 Immunofluorescence and confocal microscopy

Cells were grown on coverslips, and then fixation with 4% paraformaldehyde (15 min, room temperature), permeabilization with 0.1% Triton X-100 in PBS (10 min), and blocking with 5% bovine serum albumin (BSA) in PBS were carried out for 1 h at room temperature. Primary antibodies anti-NEDD4 (Santa Cruz Biotechnology, sc-518160; 1:100) and anti-AQP5 (Abcam, ab78486; 1:50) were then applied overnight at 4 °C. Secondary antibodies were applied next: Alexa Fluor^®^ 488 anti-rabbit (Invitrogen, A21206; 1:500) and Alexa Fluor^®^ 594 anti-mouse (Invitrogen, A21203; 1:500). Incubation with secondary antibodies was carried out in the dark for 1 h at room temperature. Coverslips were washed with PBS (three times, 5 min each). Mounting was performed with ProLong Gold Antifade Mountant containing DAPI (Invitrogen, 62248) to label nuclei. Co-localization of the two proteins was observed and analyzed using a confocal laser scanning microscope (Leica Microsystems, TCS SP8).

### 2.5 Cycloheximide (CHX) chase assay

To analyze the stability of AQP5 protein, HNEpCs were first sensitized with HDM extract (100 μg/mL) for 24 h to establish the *in vitro* allergic rhinitis model. Subsequently, cells were transfected with siNEDD4 or ovNEDD4 plasmid for 48 h to allow sufficient protein expression. Subsequently, cells were treated with CHX (10 µg/mL; Sigma-Aldrich, C4859) and harvested at 0, 6, 12, and 24 h post-CHX treatment. To examine the AQP5 protein, a Western blot (WB) was performed, and then Fiji (ImageJ 1.54p, NIH) was used to measure the intensity of the protein band and draw a degradation curve for calculating the half-life of the protein.

### 2.6 Quantitative real-time PCR (qPCR)

Total RNA was extracted from HNEpCs and nasal mucosal tissue using TRIzol reagent (Invitrogen, 15596026CN) according to the manufacturer’s instructions. RNA purity and concentration were determined with a NanoDrop 2000 (Thermo Scientific, ND-2000). Following the manufacturer’s instructions for the PrimeScript RT Reagent Kit (Takara, RR037A), cDNA was synthesized from 1 µg of total RNA. Real-time quantitative PCR (qPCR) was performed using SYBR Green Premix Ex Taq II (Takara, RR820A) on a QuantStudio 6 Flex Real-Time PCR System (Applied Biosystems, 4485697). The PCR program was set as follows: initial denaturation at 95 °C for 30 seconds; followed by 40 cycles of 95 °C for 5 seconds and 60 °C for 30 seconds. Each sample was run in triplicate.

Glyceraldehyde-3-phosphate dehydrogenase (GAPDH) was used for normalization of gene expression, and the 2−ΔΔCt method was applied. Primer sequences were as follows:

AQP5 forward: 5′-TCCATTGGCCTGTCTGTCAC-3′, reverse: 5′-GTCCTCGTCAGGCTCATACG-3′
NEDD4 forward: 5′-GCTGGAAGCGTTCGGAAATG-3′, reverse: 5′-GGTGCTGCTGAGGATGAACT-3′
GAPDH forward: 5′-GCACCGTCAAGGCTGAGAAC-3′, reverse: 5′-TGGTGAAGACGCCAGTGGA-3′

### 2.7 Ubiquitination assay

HNEpCs were co-transfected with HA-tagged ubiquitin, Myc-tagged wild-type AQP5, and FLAG-NEDD4. At 48 h following transfection, cells were incubated with 20 μM MG132 (a proteasome inhibitor; Selleck Chemicals, S2619) for 6 h to suppress proteasome enzymatic activity. Cell lysis was performed using denaturing lysis buffer (50 mM Tris-HCl, pH 7.5, 150 mM NaCl, 1% SDS, 1 mM EDTA, supplemented with protease inhibitor cocktail and 10 mM N-ethylmaleimide), followed by boiling for 10 min. Cell lysates were diluted tenfold using NETN buffer, then processed for immunoprecipitation utilizing anti-Myc magnetic microbeads (Beyotime, P2118). An anti-HA antibody (Sigma-Aldrich, H6908; 1:1,000 dilution for immunoblotting) was employed to perform an anti-HA immunoblot for the detection of ubiquitination on AQP5 (19).

### 2.8 Transepithelial electrical resistance (TER) measurement

HNEpCs were seeded onto Transwell inserts (0.4 μm, Corning, 3460) and cultured for 10 days to form a polarized monolayer. Transepithelial resistance (TER) was measured with a Millicell ERS-2 meter (Millipore, MERS00002). After TER was stabilized, the cells received the intervention, and TER was measured again after 24 h. The results were expressed as a percentage of the control.

### 2.9 Enzyme-linked immunosorbent assay (ELISA)

Cell culture supernatants were used to detect IL-6, IL-8, and MUC5AC levels in the supernatant by using ELISA kits for IL-6 (R&D Systems, D6050), IL-8 (R&D Systems, D8000C), and MUC5AC (R&D Systems, DY2197). The absorbance at 450 nm was measured using a microplate reader (Bio-Rad, 680XR) to draw the standard curve and calculate the sample concentration.

### 2.10 Animal model and *in vivo* interventions

Six- to eight-week-old male Sprague-Dawley (SD) rats were purchased. They were randomly divided into four groups (n=8 per group): the normal control group, the AR model group, the siNEDD4 group, and the ovAQP5 group. The rats were kept in Macrolon cages under a 12-hour light/dark cycle. The experimental protocol was approved by the Laboratory Animal Ethics Committee of Guangdong Medical University (approval number GDY2302671).

The rats in the three groups (the AR, siNEDD4, and ovAQP5 groups) were injected intraperitoneally with 100 μg ovalbumin (OVA; Sigma-Aldrich, A5503) adsorbed onto 2 mg aluminum hydroxide adjuvant (Beyotime, P2032), suspended in 0.2 mL of saline. The three groups were sensitized by injection three times on the first, seventh, and fourteenth days, respectively, and the control group rats were injected with the same amount of saline on the same days. Starting on the fifteenth day, all the rats of the three groups were subjected to daily intranasal instillation of 10 μL OVA solution (10 mg/mL in saline) for 7 consecutive days.

On the first day after OVA challenge, rats in the siNEDD4 and ovAQP5 groups were intranasally administered liposome-encapsulated NEDD4 siRNA or ovAQP5 plasmid, respectively (50 μg/rat; prepared with Invivogen lip-75), following the schedule in **Supplementary Figure S2**. This was repeated every 3 days for a total of three times. For ethical and practical reasons, and in line with the 3R (Replacement, Reduction, Refinement) principle to minimize the number of animals, vehicle-only, empty-vector, and non-targeting scrambled siRNA control groups were not included in the experimental design. While the convergent protective phenotypes produced by the two mechanistically independent interventions (siNEDD4 and ovAQP5) provide substantial internal biological consistency, the absence of these conventional controls is recognized as a limitation and is addressed in the Discussion.

### 2.11 Behavioral scoring and sample collection

Nasal symptoms (nasal scratching, 0–3 points; nasal discharge, 0–2 points) were scored daily during the final 3 days of OVA challenge as previously described (20).Each rat was anesthetized, and the nasal cavity was washed with 0.5 mL of saline to obtain nasal lavage fluid (NLF), so as to count inflammatory cells. Nasal mucosa tissue was obtained, part of which was fixed with paraformaldehyde to analyze histopathology, and the rest was frozen in liquid nitrogen to analyze molecules.

### 2.12 Histopathology

After fixation, the nasal mucosal tissue was processed for paraffin sectioning. Sections were cut at 5 μm and then stained with H&E. Under a light microscope (Olympus BX53), the sections from several samples were observed, and five high-power fields were selected. The number of infiltrating inflammatory cells in the submucosa was then counted using ImageJ.

### 2.13 Western blot analysis

Cells and tissues were lysed in RIPA Lysis Buffer (50 mM Tris-HCl, pH 7.4, 150 mM NaCl, 1% Triton X-100, 1% sodium deoxycholate, 0.1% SDS; Beyotime, P0013B), and the protein concentration was measured using a BCA Protein Assay Kit (Beyotime, P0012). An equal amount of protein (50 µg) was separated by 8-12% SDS-PAGE and then transferred onto a polyvinylidene difluoride (PVDF) membrane (Millipore, HVLP04700). The membranes were blocked with 5% skim milk, incubated overnight at 4 °C with the primary antibody, and then incubated with HRP-conjugated secondary antibody. Protein bands were visualized using an ECL kit (Thermo Scientific, 32209) and quantified by densitometry with Fiji (see Section 2.5). GAPDH was chosen as the loading control. The primary antibodies that have been used are as follows: anti-NEDD4 (Cell Signaling Technology, 2740; 1:1000), anti-AQP5 (Abcam, ab78486; 1:1000), anti-phospho-p38 (Sigma-Aldrich, SAB5701922; 1:800), anti-p38 (Abcam, ab4822; 1:1000), anti-MUC5AC (Abcam, ab198294; 1:500), anti-FLAG (Sigma-Aldrich, F7425; 1:1000), anti-Myc (Cell Signaling Technology, 2278S; 1:1000), and anti-GAPDH (Cwbiotech, CW0100M; 1:5000). Secondary antibodies combined with HRP were obtained from Beyotime (anti-rabbit IgG, A0208; anti-mouse IgG, P0946), both used at a dilution of 1:5000.

### 2.14 Statistical analysis

All quantitative data are expressed as mean ± standard deviation (SD). GraphPad Prism 8.0 statistical software was used to perform the analysis of statistics. A two-tailed unpaired Student’s t-test was used for two-group comparisons. One-way ANOVA followed by Tukey’s HSD *post hoc* test was used for multiple group comparisons. A *P*-value < 0.05 was considered statistically significant. All experimental assays were performed in triplicate with three biological replicates.

## 3 Results

### 3.1 NEDD4 interacts with and downregulates AQP5 in HDM-sensitized HNEpCs

In an immunoprecipitation-mass spectrometry (IP-MS) experiment with HDM-sensitized HNEpCs, AQP5 was identified as a potential NEDD4-interacting protein (**Figure 1A**). Confocal microscopy confirmed the co-localization of endogenous NEDD4 and AQP5 in these cells (**Figure 1B**). To verify a physical interaction, we carried out Co-IP experiments within cellular populations co-expressing FLAG-NEDD4 and Myc-AQP5, demonstrating their specific interaction (**Figure 1C**). A reciprocal experimental approach was also utilized to eliminate the possibility of non-specific cellular stress from siRNA off-target effects or transfection. All subsequent functional assays were assessed in parallel with siNEDD4 and ovAQP5. Functionally, the downregulation of NEDD4 by siRNA increased AQP5 protein levels in human HNEpCs exposed to HDM, but overexpression of NEDD4 decreased AQP5 levels significantly (**Figure 1D**). These results indicate that NEDD4 can interact with AQP5 and negatively regulate its protein expression.

**Figure 1:**
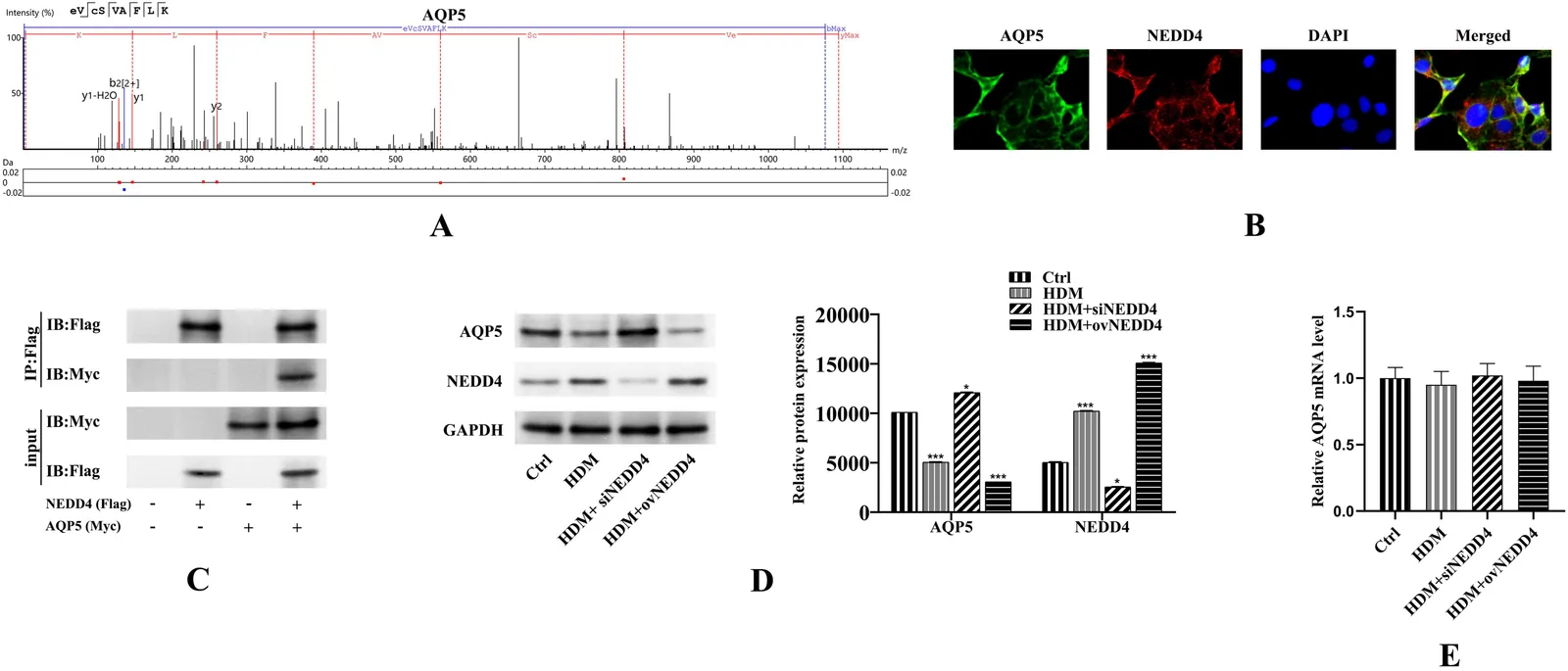
NEDD4 binds AQP5 and promotes its downregulation. **(A)** Immunoprecipitation-mass spectrometry (IP-MS) in HDM-sensitized HNEpCs transfected with FLAG-NEDD4. Immunoprecipitation was performed using an anti-FLAG antibody, and AQP5 was identified as a direct interacting partner of NEDD4. **(B)** Confocal microscopy showed co-localization of NEDD4 (red) and AQP5 (green) in HDM-sensitized HNEpCs. Scale bar: 10 μm **(C)** Co-immunoprecipitation (Co-IP) verified direct binding between FLAG-NEDD4 and Myc-AQP5 in co-transfected HNEpCs. **(D)** Western blot demonstrated that NEDD4 knockdown (siNEDD4) increased AQP5 protein levels, while NEDD4 overexpression (ovNEDD4) decreased them, in HDM-sensitized HNEpCs. **(E)** qRT-PCR revealed that modulating NEDD4 expression did not alter AQP5 mRNA levels under the same conditions as **(D)**. Data are shown as mean ± SD from three independent experiments, with each sample measured in triplicate. *P < 0.05, **P < 0.01, ***P < 0.001 compared with the control group (Ctrl). Ctrl, untreated control; HDM, house dust mite-sensitized HNEpCs; HDM + siNEDD4, HDM-sensitized HNEpCs transfected with siNEDD4; HDM + ovNEDD4, HDM-sensitized HNEpCs transfected with NEDD4 overexpression plasmid. GAPDH acted as a normalization standard; consistent band intensities confirmed equal protein loading.

Notably, the suppression of NEDD4 did not alter the levels of AQP5 mRNA (**Figure 1E**), serving as a significant internal control. This verifies that transfection did not cause general transcriptional suppression or reduced cell viability. The mismatch between stable AQP5 mRNA levels and reduced protein levels indicates post-translational regulation, consistent with the described E3 ligase activity of NEDD4. Furthermore, the consistent and opposing effects of NEDD4 knockdown and overexpression, observed simultaneously throughout our study, provide strong internal validation. These results support the conclusion that the observed phenotypes are due to the specific regulation of the NEDD4-AQP5 axis and not due to non-specific transfection artifacts.

### 3.2 NEDD4 promotes ubiquitination of AQP5 at K257

NEDD4, an E3 ubiquitin ligase, might be a potential mediator of ubiquitin-dependent degradation of AQP5. We assessed the stability of the AQP5 protein through CHX chase experiments in HDM-primed HNEpCs. In the CHX chase assay, transfection with siNEDD4 caused a modest but significant reduction in NEDD4 protein levels at 12 h and 24 h (by approximately 18.3% and 38.6%, respectively; both P < 0.05), along with a marked prolongation of AQP5 half-life (**Figure 2A**). Consistently, AQP5 was significantly more stable in the siNEDD4 group than in the HDM group at 24 h post-CHX treatment (P < 0.001), supporting that reduced NEDD4 activity prolongs AQP5 half-life. In contrast, NEDD4 overexpression accelerated AQP5 degradation, significantly reducing AQP5 levels at 6 h and 12 h post-CHX (both P < 0.01 vs. HDM). Together, these results indicate that downregulation of NEDD4 prolongs AQP5 half-life, whereas overexpression shortens it. Degradation of AQP5 was inhibited via proteasome blocker MG132, but not the lysosomal suppressor chloroquine (CQ) (**Figure 2B**), suggesting that AQP5 undergoes degradation mainly via the proteasome cascade. Accordingly, downregulation of NEDD4 notably lowered the polyubiquitination of AQP5 (**Figure 2C**). Quantification of the ubiquitination signals showed no significant difference between the siNEDD4 and ovNEDD4 groups.

**Figure 2:**
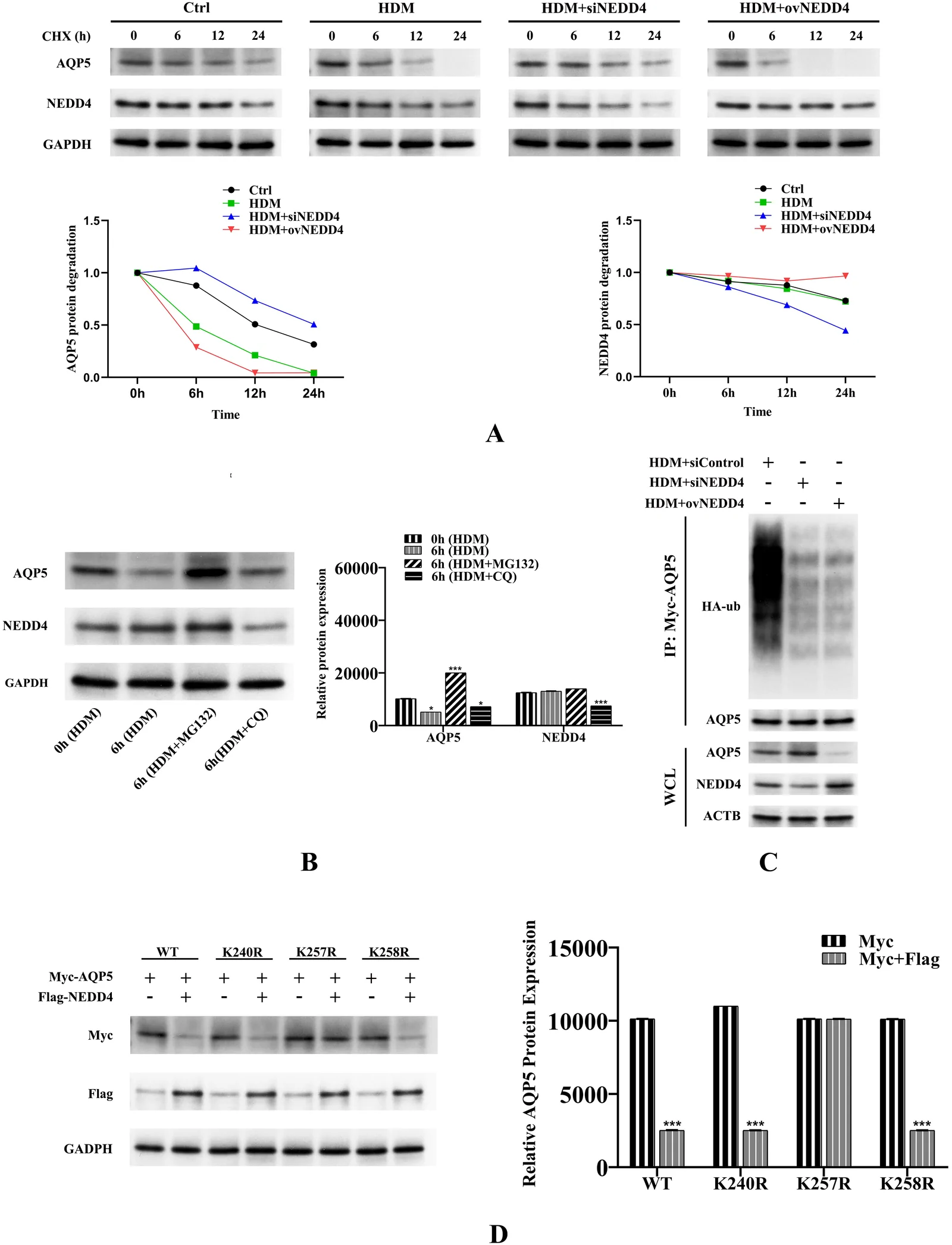
NEDD4 targets AQP5 for ubiquitin-proteasome degradation at K257. **(A)** HDM-sensitized HNEpCs were transfected with siNEDD4 or ovNEDD4 plasmid for 72 h, then exposed to 100 μg/mL cycloheximide (CHX) over time points of 0, 6, 12, and 24 h. AQP5 protein levels were detected by Western blot and quantified by ImageJ. siNEDD4 prolonged AQP5 protein half-life, whereas ovNEDD4 shortened it. **(B)** HDM-sensitized HNEpCs were exposed to MG132 (proteasome antagonist) or chloroquine (CQ, lysosomal antagonist) for 6 h. AQP5 expression was detected by Western blot. Only MG132 blocked AQP5 degradation. **(C)** HNEpCs were transfected with siNEDD4 or ovNEDD4, pretreated with MG132, then lysed in 1% SDS denaturing buffer. AQP5 ubiquitination was detected by anti-Myc immunoprecipitation (IP: Myc-AQP5) followed by anti-HA immunoblotting (IB: HA-ub) **(D)** HDM-sensitized HNEpCs were co-transfected with Myc-AQP5 (WT or K240R/K257R/K258R mutants) and FLAG-NEDD4 plasmid for 24 h. AQP5 protein levels were measured via Western blotting. Only the K257R mutant resisted NEDD4-mediated degradation. Data are shown as mean ± SD from three independent experiments, with each sample measured in triplicate. *P < 0.05, **P < 0.01, ***P < 0.001 vs. Ctrl group. Ctrl, untreated control; HDM, house dust mite-sensitized HNEpCs; HDM + siNEDD4, HDM-sensitized HNEpCs transfected with siNEDD4; HDM + ovNEDD4, HDM-sensitized HNEpCs transfected with NEDD4 overexpression plasmid. GAPDH served as a reference marker; consistent GAPDH band densities confirmed equal protein loading.

In order to identify the critical ubiquitination sites, we created lysine-to-arginine mutants of AQP5 at three candidate ubiquitination sites (K240R, K257R, K258R). Of these, only the K257R mutant was resistant to NEDD4-induced degradation (**Figure 2D**), which identified K257 as the main ubiquitin acceptor site. Collectively, these results indicate that NEDD4 mediates AQP5 proteasomal degradation through selective ubiquitination on K257.

### 3.3 Targeting the NEDD4-AQP5 axis restores barrier function and attenuates inflammation

HDM stimulation over a 24-hour period caused hypersecretion of MUC5AC as well as reduced TER of HNEpCs (**Figures 3A, B**). NEDD4 silencing or AQP5 overexpression reversed HDM-induced MUC5AC overproduction and restored TER to near-normal levels. The exposure to HDM also stimulated the release of pro-inflammatory mediators IL-6 and IL-8, but their secretion was significantly inhibited by either NEDD4 silencing or AQP5 overexpression (**Figures 3C, D**). This evidence shows that the NEDD4-AQP5 pathway exerts a critical function in modulating nasal mucosal epithelial barrier homeostasis and inflammation within HDM-sensitized HNEpCs. The fact that both NEDD4 silencing and AQP5 restoration have convergent protective phenotypes does not suggest off-target or vector-specific effects.

**Figure 3:**
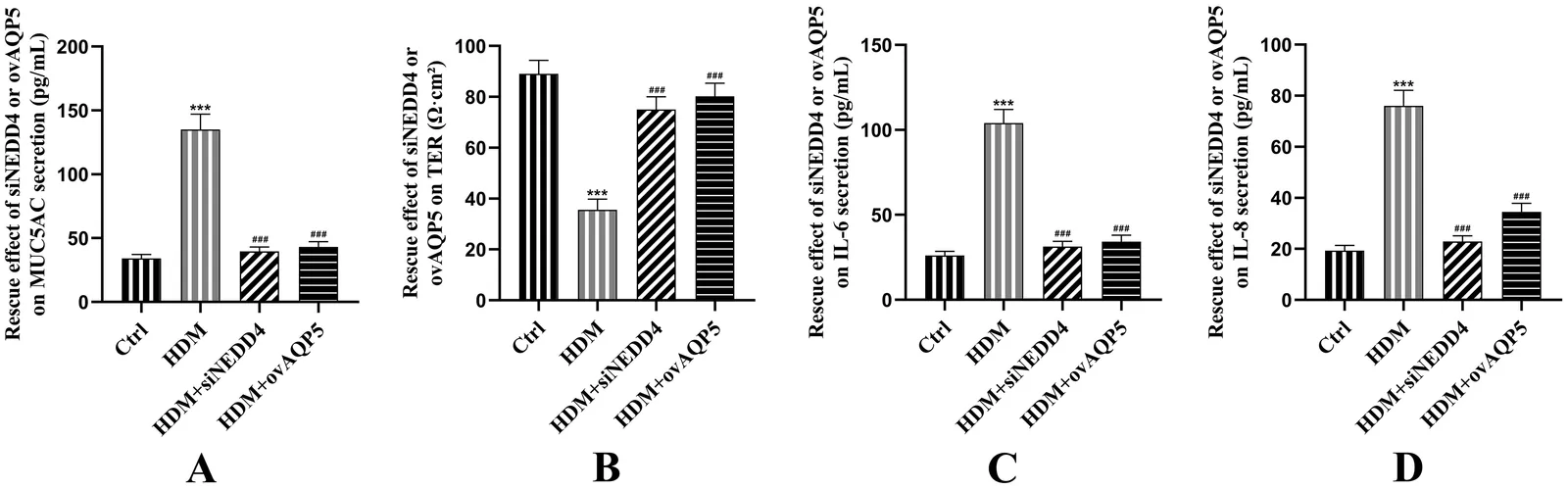
Disruption of the NEDD4-AQP5 axis impairs barrier function and promotes cytokine release. **(A)** MUC5AC amounts (pg/mL) in cellular culture supernatants were assayed with ELISA across various treatment groups. **(B)** TER (Ω·cm²) of HNEpC monolayers was measured utilizing a Millicell ERS-2 system across the specified groups. **(C, D)** The amounts of IL-6 (pg/mL) and IL-8 (pg/mL) in cellular culture supernatants were determined via ELISA. Data are shown as mean ± SD from three independent experiments, with each sample measured in triplicate. *P < 0.05, **P < 0.01, ***P < 0.001 vs. Ctrl group; ^#^P < 0.05, ^##^P < 0.01, ^###^P < 0.001 compared with the HDM group. Ctrl, untreated control; HDM, house dust mite-sensitized HNEpCs; HDM + siNEDD4, HDM-sensitized HNEpCs transfected with siNEDD4; HDM + ovAQP5, HDM-sensitized HNEpCs transfected with AQP5 overexpression plasmid.

### 3.4 The NEDD4-AQP5 axis disrupt barrier homeostasis via p38 MAPK activation

In order to determine the downstream signaling pathway, we tested the phosphorylation of several key inflammatory signaling molecules in HDM-stimulated HNEpCs by Western blot, including c-Jun N-terminal kinase (JNK), extracellular signal-regulated kinase (ERK), signal transducer and activator of transcription 6 (STAT6), Akt (protein kinase B), and nuclear factor-κB p65 (p65). As NEDD4 is an E3 ubiquitin ligase and lacks intrinsic kinase activity, it cannot directly phosphorylate any of these molecules. Among the tested proteins, p38 MAPK phosphorylation showed the most significant increase upon HDM exposure (**Figure 4A**). Gain- and loss-of-function experiments revealed that overexpression of NEDD4 was associated with elevated levels of HDM-induced p38 MAPK phosphorylation, whereas knockdown of NEDD4 or overexpression of AQP5 attenuated that p38 MAPK phosphorylation (**Figure 4B**). To confirm the functional role of p38 MAPK, we then applied the specific p38 MAPK inhibitor SB203580 (10 μM, 1h). The p38 phosphorylation observed in HDM-treated or NEDD4-overexpressing cells was completely inhibited by SB203580 (**Figure 4C**). Inhibiting p38 effectively counteracted the MUC5AC hypersecretion induced by HDM or NEDD4 overexpression (**Figure 4D**). This evidence indicates that the NEDD4-AQP5 axis aggravates AR-mediated barrier dysfunction and inflammation indirectly through p38 MAPK activation.

**Figure 4:**
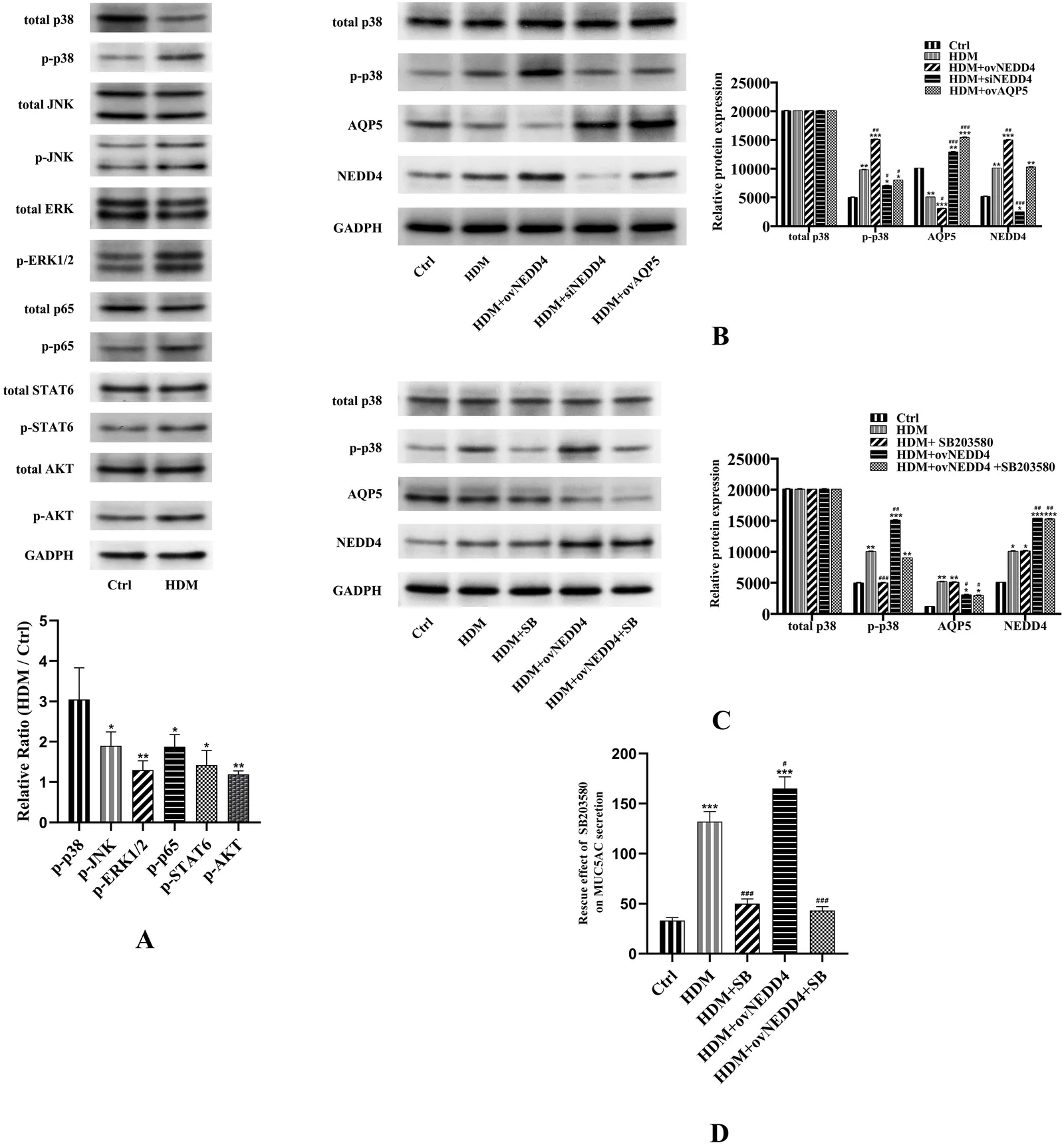
NEDD4-mediated AQP5 ubiquitination indirectly associates with p38 MAPK activation and MUC5AC hypersecretion. **(A)** Western blot analysis measuring phosphorylated and total protein species of p38, JNK, ERK, STAT6, AKT, and p65 in control and HDM-sensitized HNEpCs. HDM exposure increased the phosphorylation of all examined proteins to varying extents, with the most robust elevation observed for p38 MAPK. **(B)** Western blot analysis of p38 phosphorylation in HDM-sensitized HNEpCs. NEDD4 overexpression enhanced p-p38 levels, whereas NEDD4 knockdown or AQP5 overexpression blunted this response. **(C)** The p38-specific inhibitor SB203580 blocked p38 phosphorylation triggered by either HDM stimulation or NEDD4 overexpression in HNEpCs. **(D)** Quantitative analysis confirmed that SB203580 reversed HDM- or NEDD4 overexpression-induced MUC5AC hypersecretion. Data are shown as mean ± SD from three independent experiments, with each sample measured in triplicate. *P < 0.05, **P < 0.01, ***P < 0.001 vs. Ctrl group; ^#^P < 0.05, ^##^P < 0.01, ^###^P < 0.001 compared with the HDM group. Ctrl, untreated control; HDM, house dust mite-sensitized HNEpCs; HDM + siNEDD4, HDM-sensitized HNEpCs transfected with siNEDD4; HDM + ovAQP5, HDM-sensitized HNEpCs transfected with ovAQP5; HDM + SB, HDM-sensitized HNEpCs treated with SB203580; HDM + ovNEDD4, HDM-sensitized HNEpCs transfected with ovNEDD4; HDM + siNEDD4 + SB, HDM-sensitized HNEpCs transfected with siNEDD4 and treated with SB203580. GAPDH acted as a normalization standard; consistent GAPDH band intensities confirmed equal protein loading.

### 3.5 IL-13 drives NEDD4 upregulation in HNEpCs

To determine which Th2 cytokines upregulated NEDD4 in the AR inflammatory microenvironment, HNEpCs were subjected to sequential doses of IL-4 or IL-13. These two cytokines increased NEDD4 protein expression in a dose-dependent manner, with IL-13 being much more effective than IL-4 (**Figure 5A**). Quantitative PCR (qPCR) examinations demonstrated that IL-13 also elicited a stronger response in terms of NEDD4 mRNA levels than IL-4 (**Figure 5B**). Neutralizing IL-13 in HDM-sensitized HNEpCs significantly inhibited NEDD4 upregulation (**Figure 5C**). The data imply that IL-13 acts as the primary upstream signaling molecule driving NEDD4 overexpression in nasal epithelial cells, linking Th2-type inflammation to the NEDD4-AQP5 regulatory axis in AR.

**Figure 5:**
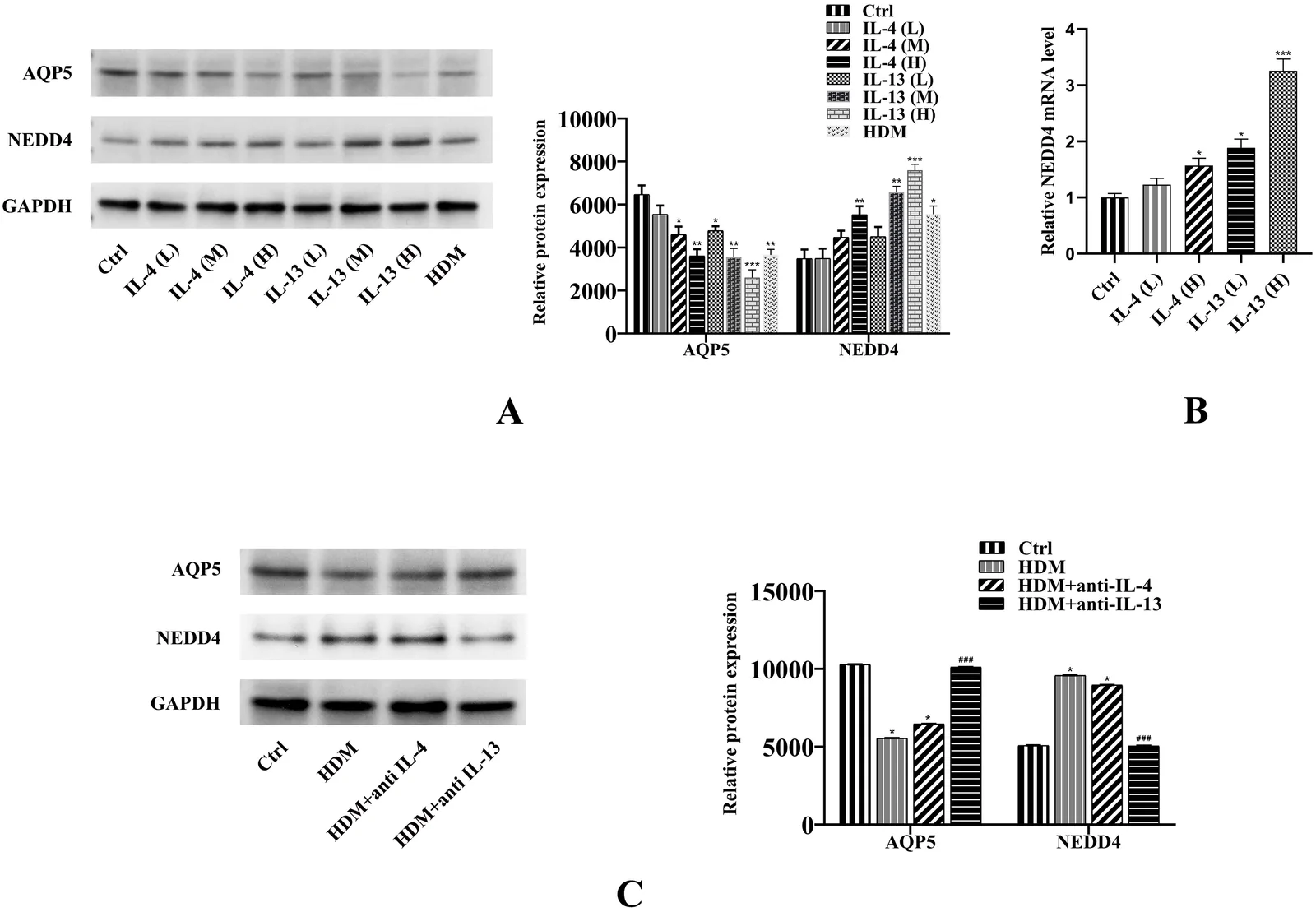
IL-13, rather than IL-4, potently induces NEDD4 expression. **(A)** Serial concentrations of IL-4 or IL-13 dose-dependently upregulate NEDD4 protein expression in HNEpCs, with IL-13 exerting a stronger effect as determined by Western blot. **(B)** IL-13 induces a more robust increase in NEDD4 mRNA levels than IL-4 in HNEpCs (qPCR). **(C)** Neutralization of IL-13 markedly suppresses NEDD4 upregulation in HDM-sensitized HNEpCs. Data are shown as mean ± SD from three independent experiments, with each sample measured in triplicate. *P < 0.05, **P < 0.01, ***P < 0.001 vs. Ctrl group; ^&^P < 0.05, ^&&^P < 0.01, ^&&&^P < 0.001 vs. the IL-4 (L) group; ^#^P < 0.05, ^##^P < 0.01, ^###^P < 0.001 vs. the HDM group. Ctrl, untreated control; L, M, H: low (10 ng/mL), medium (20 ng/mL), and high (50 ng/mL) levels of IL-4 or IL-13, respectively; HDM, house dust mite-sensitized HNEpCs; HDM + anti-IL-4, HDM-sensitized HNEpCs treated with IL-4 neutralizing antibody; HDM + anti-IL-13, HDM-sensitized HNEpCs treated with IL-13 neutralizing antibody. GAPDH acted as a normalization standard; consistent GAPDH band intensities confirmed equal protein loading.

### 3.6 *In vivo* validation of the NEDD4-AQP5 axis in an OVA-induced AR rat model

In order to elucidate the *in vivo* significance of the NEDD4-AQP5 axis, we created a rat model of OVA-evoked allergic rhinitis. Immunoblot detection on nasal mucosal samples showed that AR induction caused a tremendous increase in NEDD4 and p-p38 expression with a significant decrease in AQP5 protein (**Figure 6A**). Interestingly, intranasal administration of siNEDD4 not only inhibited the expression of NEDD4 but also recovered AQP5 protein levels and reduced p38 MAPK activation. Similarly, ovAQP5 could restore AQP5 levels without changing NEDD4 levels.

**Figure 6:**
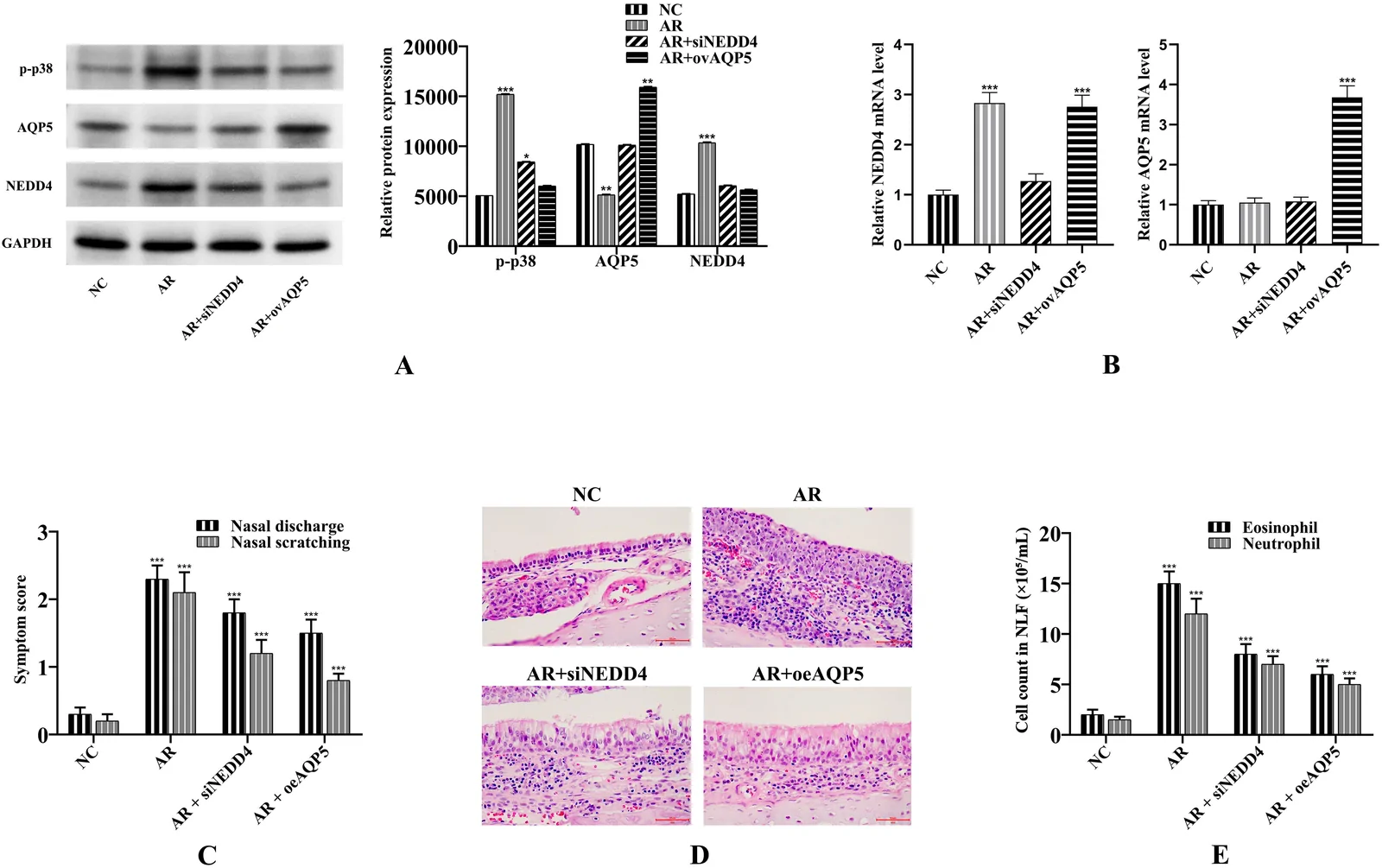
Targeting NEDD4 or restoring AQP5 alleviates allergic inflammation *in vivo.* **(A)** Western blot and quantitative measurement of p-p38, AQP5, and NEDD4 expression in rat nasal mucosa. **(B)** Real-time qPCR assays for NEDD4 and AQP5 mRNA expression in nasal mucosa. **(C)** Nasal discharge and nasal scratching symptom scores in rats. **(D)** H&E staining of rat nasal mucosa (scale bar = 50 μm), showing inflammatory infiltration and edema. **(E)** Eosinophil and neutrophil counts in NLF of rats. Data are shown as mean ± SD from three independent experiments, with each sample measured in triplicate. *P < 0.05, **P < 0.01, ***P < 0.001 compared with NC group. NC, Normal control; AR, allergic rhinitis model; AR + siNEDD4: AR model with intranasal administration of siNEDD4; AR + ovAQP5: AR model with intranasal administration of ovAQP5. GAPDH acted as a normalization standard; consistent GAPDH band intensities confirmed equal protein loading.

While ovAQP5 increased AQP5 mRNA expression, siNEDD4 did not affect AQP5 transcript levels (**Figure 6B**), indicating that NEDD4 functions as a repressive modulator of AQP5 at the post-translational level *in vivo*. This functional recovery also correlated with significant improvement of allergic symptomatology, which was measured by reduced nasal discharge and scratching rate (**Figure 6C**). H&E histological section staining also supported the fact that NEDD4 knockdown or AQP5 overexpression significantly decreased inflammatory cell infiltration and mucosal edema (**Figure 6D**). The numbers of eosinophils and neutrophils in the NLF were also reduced compared with those in the AR group after treatment (**Figure 6E**).

The above *in vivo* results support the results obtained *in vitro* and strengthen the hypothesis that the NEDD4-AQP5 axis is related to AR.

## 4 Discussion

We report a novel regulatory pathway based on NEDD4 and AQP5 that triggers nasal epithelial barrier breakdown and exaggerated inflammatory response in AR, leading to inflammation amplification. Clinical investigations have shown that AQP5 is markedly reduced in nasal tissue of AR patients, and this drop correlates with decreased barrier integrity and symptom severity (7). However, the post-translational pathways underlying AQP5 depletion under such circumstances have not been specified. We demonstrate here that NEDD4, a HECT-family E3 ubiquitin ligase found at higher levels in the inflammatory environment of AR nasal epithelia, can directly bind to AQP5 and ubiquitinate it at lysine 257 (K257), directing it to proteasome-mediated degradation without altering AQP5 transcription. Water transport is impaired by NEDD4-mediated AQP5 deficiency. Downstream activation of the p38 MAPK signaling pathway is linked to this deficiency. The precise molecular link between AQP5 loss and p38 phosphorylation remains to be fully elucidated. The above findings deepen the understanding of the post-translational regulation mechanism of AQP5 and indicate that the NEDD4-AQP5 axis is involved in the pathogenesis of AR, which may be conducive to the next stage of treatment.

This study demonstrates that NEDD4 mediates the ubiquitin-dependent degradation of AQP5, offering a new paradigm for PTM-driven regulation of the nasal mucosal epithelial barrier. Ubiquitination regulates protein stability and is closely connected with epithelial barrier function and immune regulation (21–23). NEDD4 has been found to act upon various substrates through selective ubiquitination (24, 25), and our findings show that NEDD4 binds directly to AQP5 in HDM-sensitized human nasal epithelial cells, and this interaction is identified by co-immunoprecipitation, mass spectrometry, and confocal imaging. Through site-directed mutagenesis of three candidate lysine residues (K240, K257, and K258) on AQP5, we identified that only the K257R mutant—in which lysine 257 was substituted with arginine—was resistant to NEDD4-mediated degradation (**Figure 2D**). This resistance indicates that lysine 257 (K257) is the principal ubiquitin acceptor site through which NEDD4 targets AQP5 for proteasomal degradation. This process is analogous to the regulatory pathway described in renal epithelia concerning AQP2, where NEDD4 induces ubiquitination at the C-terminal lysine residues and degradation of AQP2 (26). In our ubiquitination assay (**Figure 2C**), AQP5 ubiquitination levels were comparable between NEDD4 knockdown and overexpression groups. This observation seems counterintuitive. However, it can be explained by the dynamic equilibrium of the ubiquitin-proteasome system. Overexpression of NEDD4 enhances AQP5 ubiquitination flux, yet the ubiquitinated proteins are rapidly degraded. When MG132 blocks degradation, the substrate pool becomes depleted, which restricts the detectable signal. In contrast, NEDD4 knockdown reduces degradation. This increases AQP5 stability and substrate availability, potentially compensating for the reduced ligase activity. The relationship between E3 ligase expression and substrate ubiquitination is not necessarily linear. Several factors—including substrate saturation, enzyme kinetics, and additional post-translational regulatory mechanisms—can influence the observed signal (13, 15). Therefore, in the presence of MG132, the ubiquitination signal reflects a complex steady-state equilibrium. It is not a simple linear readout of E3 ligase activity. This interpretation is consistent with the established dynamics of the ubiquitin-proteasome system (26). Overall, NEDD4 regulates AQP5 stability in nasal mucosa through a conserved mechanism, and this extends the relevance of the pathway to other epithelial tissues.

The integrity of the epithelial barrier is orchestrated by multiple PTMs acting in concert. Steelant et al. demonstrated that HDAC activity is aberrantly elevated in nasal epithelial cells from individuals with AR, and that this heightened activity epigenetically suppresses the expression of tight junction proteins, including occludin and ZO-1, thereby compromising barrier function (10). This transcriptional mode of regulation is mediated by acetylation, contrasting sharply with the post-translational mechanism described here. In that mechanism, AQP5 is ubiquitinated by NEDD4 at lysine 257 and subsequently targeted for proteasomal degradation. These findings suggest that PTMs cooperate during AR-associated barrier disruption, at both the transcriptional (acetylation) and post-translational (ubiquitination) levels. Furthermore, Liang et al. revealed a critical role for SUMOylation in the airway epithelial barrier: chromobox 4 (CBX4)-mediated SUMOylation of β-catenin does not promote its degradation, but instead alters its subcellular distribution and activates Wnt/β-catenin signaling (8). This outcome is fundamentally distinct from the fate of AQP5 following ubiquitination at the K257 residue, which is targeted for direct proteasomal degradation, underscoring how different types of PTMs and distinct modification sites differentially govern the stability and function of the substrate protein. Together with these studies, our findings delineate an intricate and highly coordinated PTM regulatory network-comprising ubiquitination, SUMOylation and acetylation-that plays a central role in maintaining nasal epithelial barrier homeostasis and driving the pathogenesis of AR.

Impairment of nasal epithelial barrier performance in AR is characterized by elevated permeability, excess mucus production, and disrupted junctional structure (7, 27). AQP5 is the main water channel of nasal epithelia that maintains the barrier integrity by maintaining the cellular water flux and mucus hydration (6). Our data show that HDM increases NEDD4 levels, leading to AQP5 degradation, a progressive decline in TER, and excessive MUC5AC secretion. Reduction of NEDD4 or restoration of AQP5 mitigated these abnormalities, confirming the protective function of AQP5 in nasal barrier stability (7). In addition to its role in barrier function, the NEDD4-AQP5 pathway also regulates the release of IL-8 and IL-6, indicating its involvement in both inflammation and epithelial barrier structure.

The p38 MAPK pathway is one of the main regulators of inflammatory signaling and has also been associated with barrier damage and excess production of mucus in AR (17, 28). Our data place p38 MAPK as an important downstream effector of the NEDD4-AQP5 axis. Following HDM treatment, NEDD4 upregulation leads to AQP5 degradation, which in turn is associated with increased p38 phosphorylation; conversely, overexpression of AQP5 prevents this increase. In this case, the barrier is destroyed and the secretion of MUC5AC increases, but the use of SB203580 can effectively inhibit p38. These findings reveal a functional signaling cascade in which NEDD4-mediated degradation of AQP5 is linked to downstream activation of p38 MAPK, thereby connecting dysregulation of aquaporins to mucosal inflammation and extending previous reports that p38 MAPK induces mucin transcription and inflammatory mediator release in AR (29). The findings reported by Liu et al. in a lung barrier injury model closely parallel our observations: the toxin MC-LR enhances claudin-1 ubiquitination to promote its degradation, resulting in pulmonary epithelial disruption and pneumonia (9). Collectively, ubiquitin-proteasome pathway-mediated degradation of key barrier proteins (such as AQP5, Claudin-1) may represent a universal core mechanism through which exogenous insults (allergens, toxins) and endogenous inflammatory signals (e.g., IL-13) inflict barrier damage. As an E3 ubiquitin ligase, NEDD4 does not directly phosphorylate p38. We hypothesize that AQP5 degradation and the consequent disruption of cellular water transport or barrier integrity may generate stress signals-such as osmotic stress or secondary inflammatory mediators-that in turn activate the p38 MAPK cascade. The precise molecular events linking AQP5 loss to p38 phosphorylation warrant further investigation.

AR development is characterized by Th2-type inflammation, with IL-4 and IL-13 as the leading effector cytokines (30, 31). In the study, it was found that IL-13 is the main upstream activator of NEDD4 and is more active than IL-4 in inducing NEDD4 expression in nasal epithelial cells at the protein and mRNA levels. When IL-13 was neutralized in HDM-sensitized cells, NEDD4 expression was not increased, and AQP5 expression remained relatively unchanged. In this case, a self-reinforcing cycle can be formed: after being stimulated by allergens, IL-13 is released and NEDD4 is activated. After a period of time, NEDD4 degrades AQP5, leading to loss of barrier function and increased allergen entry, resulting in Th2 polarization. The axis connects the activation of Th2 immunity and the breakdown of the epithelial barrier, offering a combined overview of AR pathophysiology.

Several limitations of the present study should be acknowledged. First, in both the *in vitro* and *in vivo* experiments, standard controls-specifically, empty-vector controls for overexpression and non-targeting scrambled siRNA controls for knockdown-were not included. While the reciprocal rescue observed between NEDD4 loss-of-function and AQP5 gain-of-function provides strong internal biological consistency and substantially mitigates concerns about off-target effects or transfection-related artifacts, it does not fully replace these conventional controls. The possibility cannot be completely excluded that certain non-specific effects contributed to the observed phenotypes. Second, the animal experiments lacked vehicle-only and empty-vector control groups. The 3R principle guided our decision to minimize the number of animals used. Potential nonspecific effects from the lipid-based delivery system or the plasmid backbone cannot be definitively excluded. The convergent phenotypes produced by two mechanistically independent interventions (siNEDD4 and ovAQP5) argue against a significant contribution of such artifacts. These *in vivo* conclusions would be further strengthened by confirmatory studies incorporating appropriate vehicle and empty-vector controls.

Taken together, our observations identify that the NEDD4-AQP5 axis is one of the driving factors for epithelial barrier dysfunction and inflammation in AR. Beyond this work, several questions remain open. Whether NEDD4 also disrupts barrier integrity by targeting tight junction components, such as ZO-1 or occludin, needs to be determined. AQP5 may be subject to additional post-translational modifications, including phosphorylation or SUMOylation, which may crosstalk with ubiquitination to modulate AQP5 stability and function. Exploring these issues through proteomic and functional approaches could further illuminate the regulatory networks governing nasal epithelial homeostasis in allergic rhinitis.

This work provides a new direction for AR research. In nasal epithelial cells, IL-13 upregulates NEDD4 expression. AQP5 is subsequently ubiquitinated at K257 and degraded by the proteasome, leading to reduced AQP5 levels and impaired barrier function. Concurrently, p38 MAPK activation triggers excessive mucus secretion and severe inflammation. These data help clarify the epithelial barrier alterations in AR and elucidate the interplay between AQP5 and NEDD4 in AR pathogenesis. The inverse correlation between the two molecules can be validated by proteomic or immunoblotting analysis of clinical samples.

## Data Availability

The raw data supporting the conclusions of this article will be made available by the authors, without undue reservation.
