# Supplementary material for: The role of NEDD4-mediated ubiquitination of AQP5 in allergic rhinitis: implications for nasal mucosal barrier function
Source: Front Immunol. 2026 Sep 17;17:1908261. doi: 10.3389/fimmu.2026.1908261 (PMC13626906; doi:10.3389/fimmu.2026.1908261)
Supplement: Supplementary file 1 [file DataSheet1.pdf]

Supplementary Figure S1. Plasmid maps of the constructs used in this study

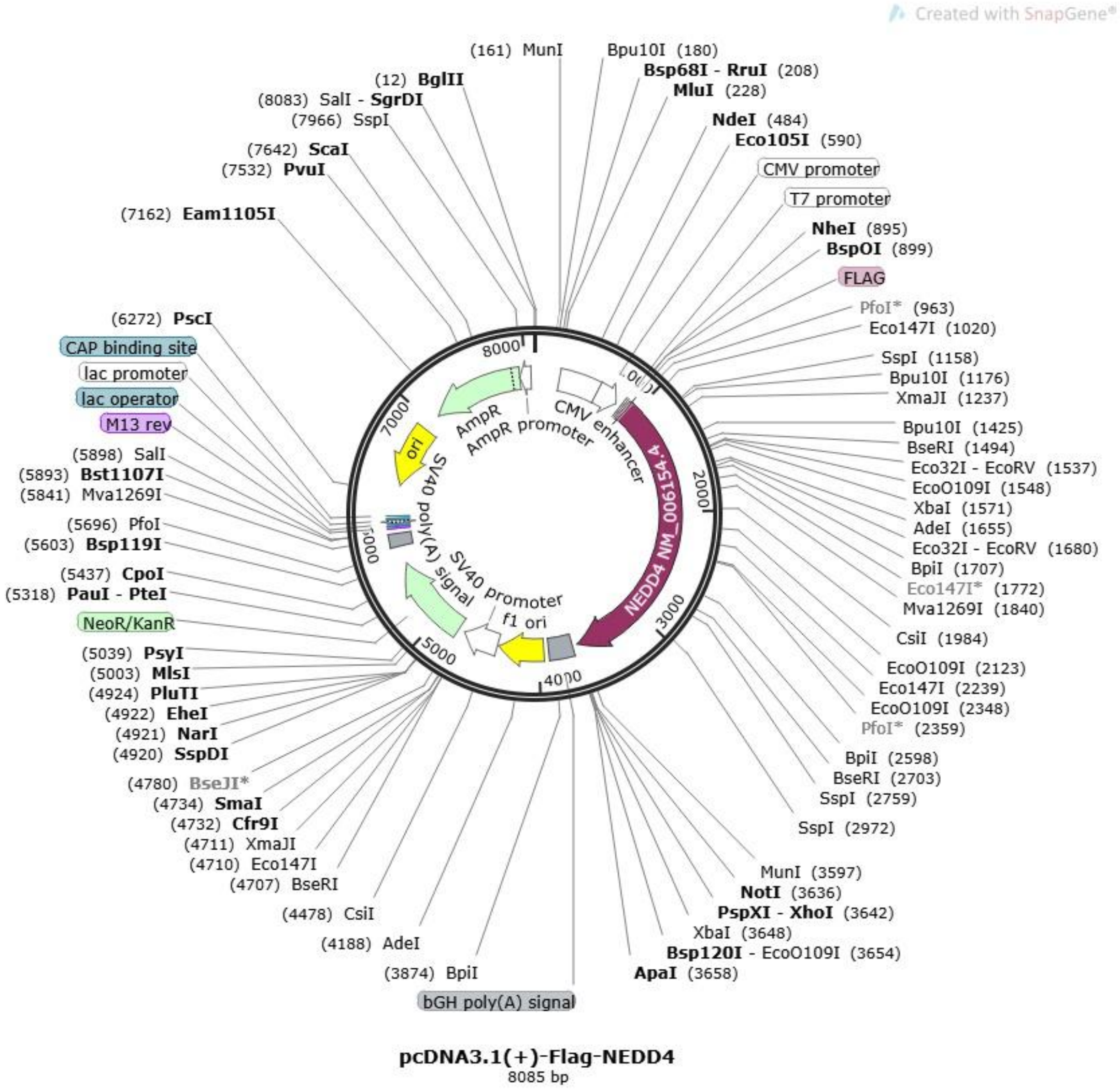

(A) Map of the pcDNA3.1-FLAG-**hNEDD4** vector (8085 bp). The construct encodes full-length human NEDD4 with an N-terminal FLAG tag.

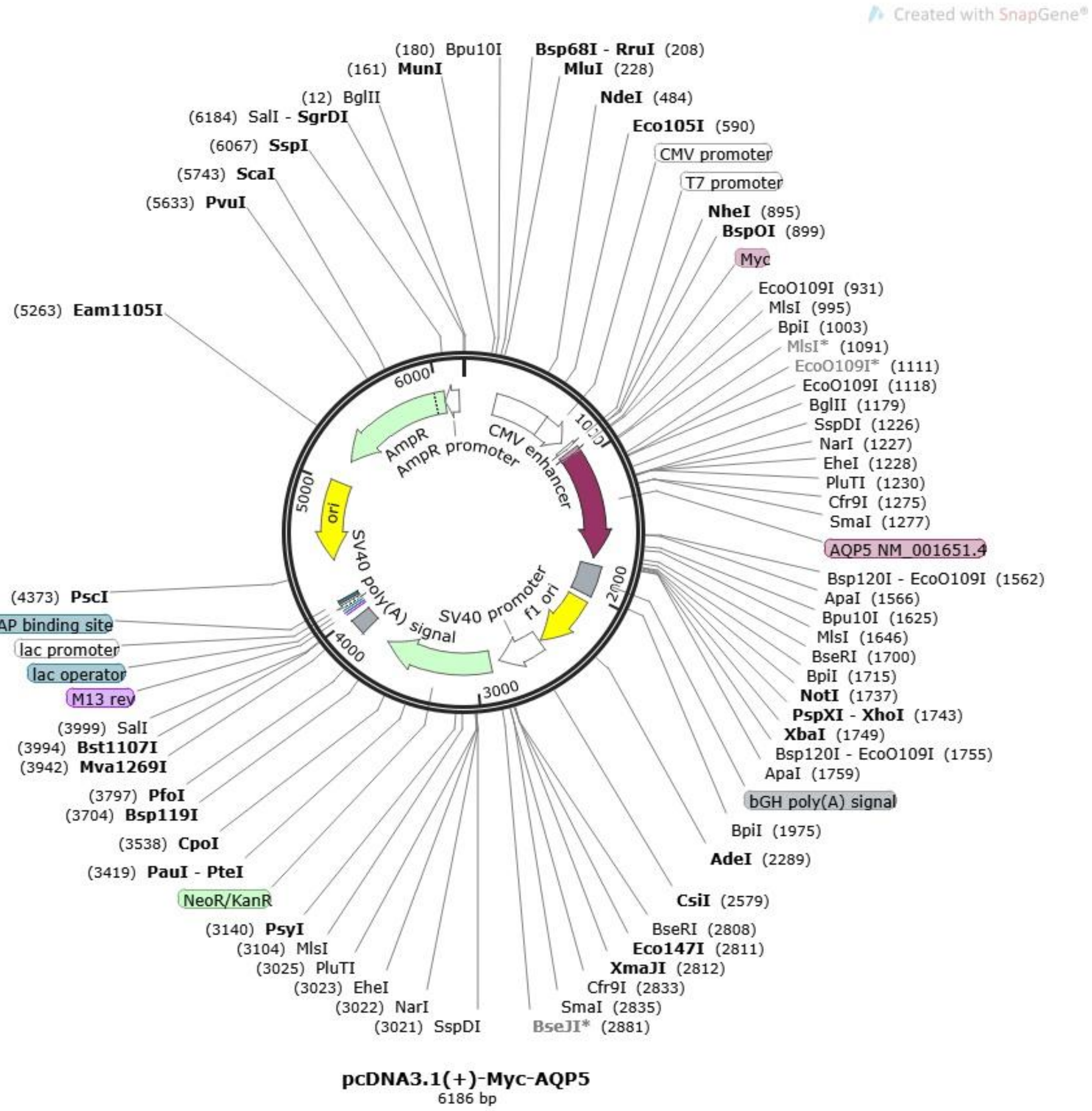

(B) Map of the pcDNA3.1-Myc-**hAQP5** wild-type vector (6186 bp). The construct encodes full-length human AQP5 with an N-terminal Myc tag.

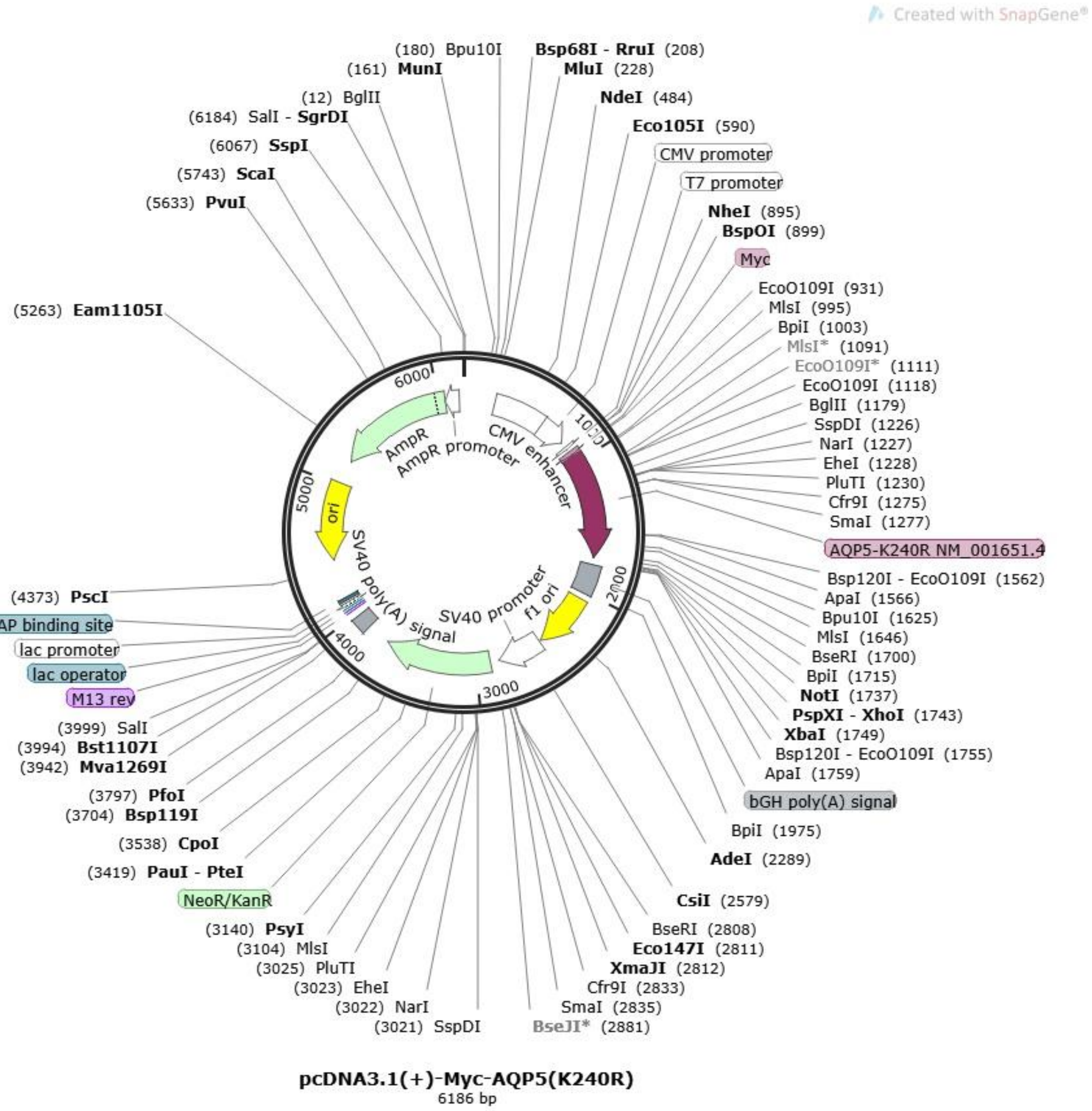

(C) Map of the pcDNA3.1-Myc-hAQP5(**K240R**) mutant vector (6186 bp). The construct encodes human AQP5 with a lysine-to-arginine mutation at position 240.

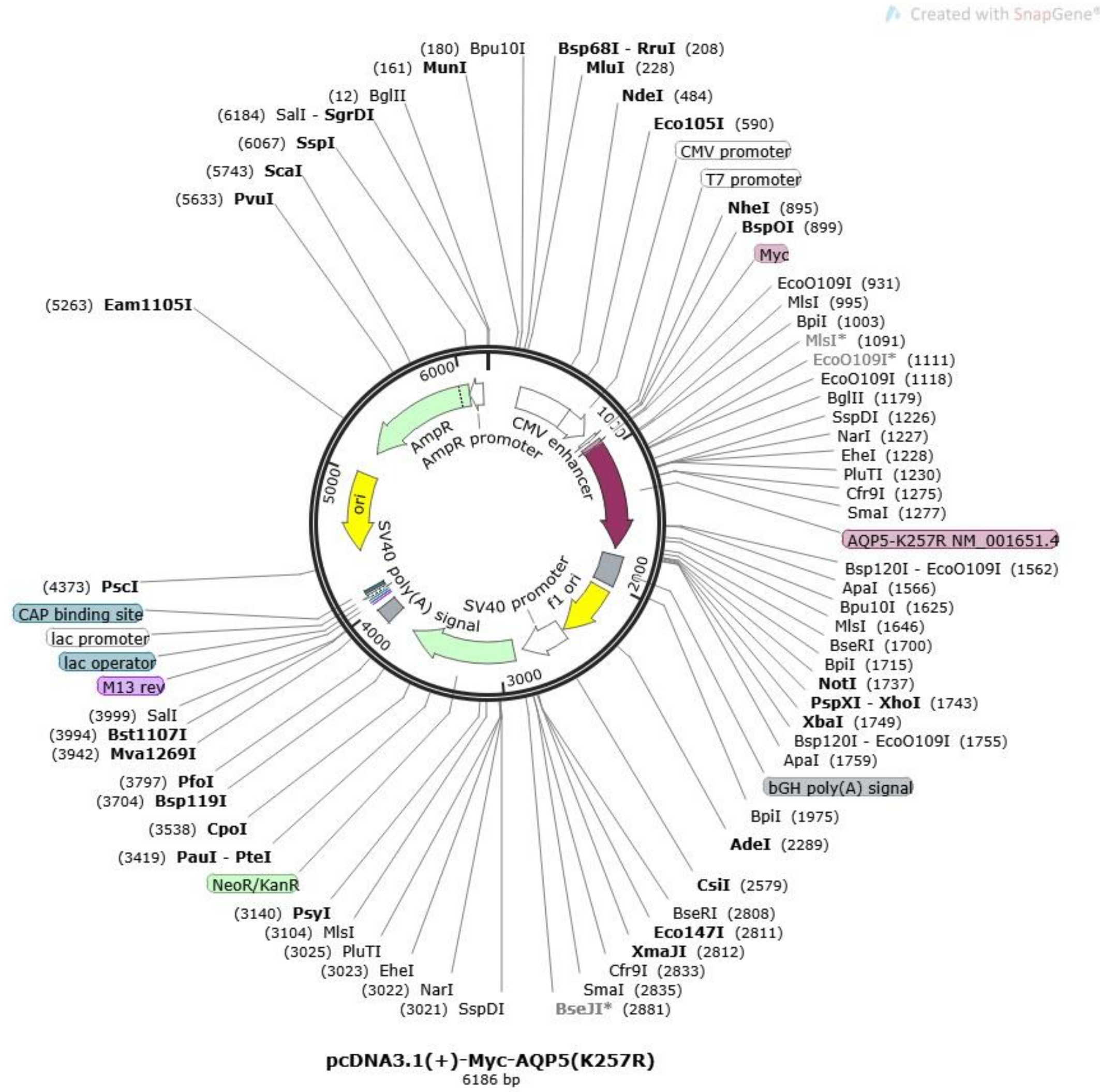

(D) Map of the pcDNA3.1-Myc-hAQP5(**K257R**) mutant vector (6186 bp). The construct encodes human AQP5 with a lysine-to-arginine mutation at position 257.

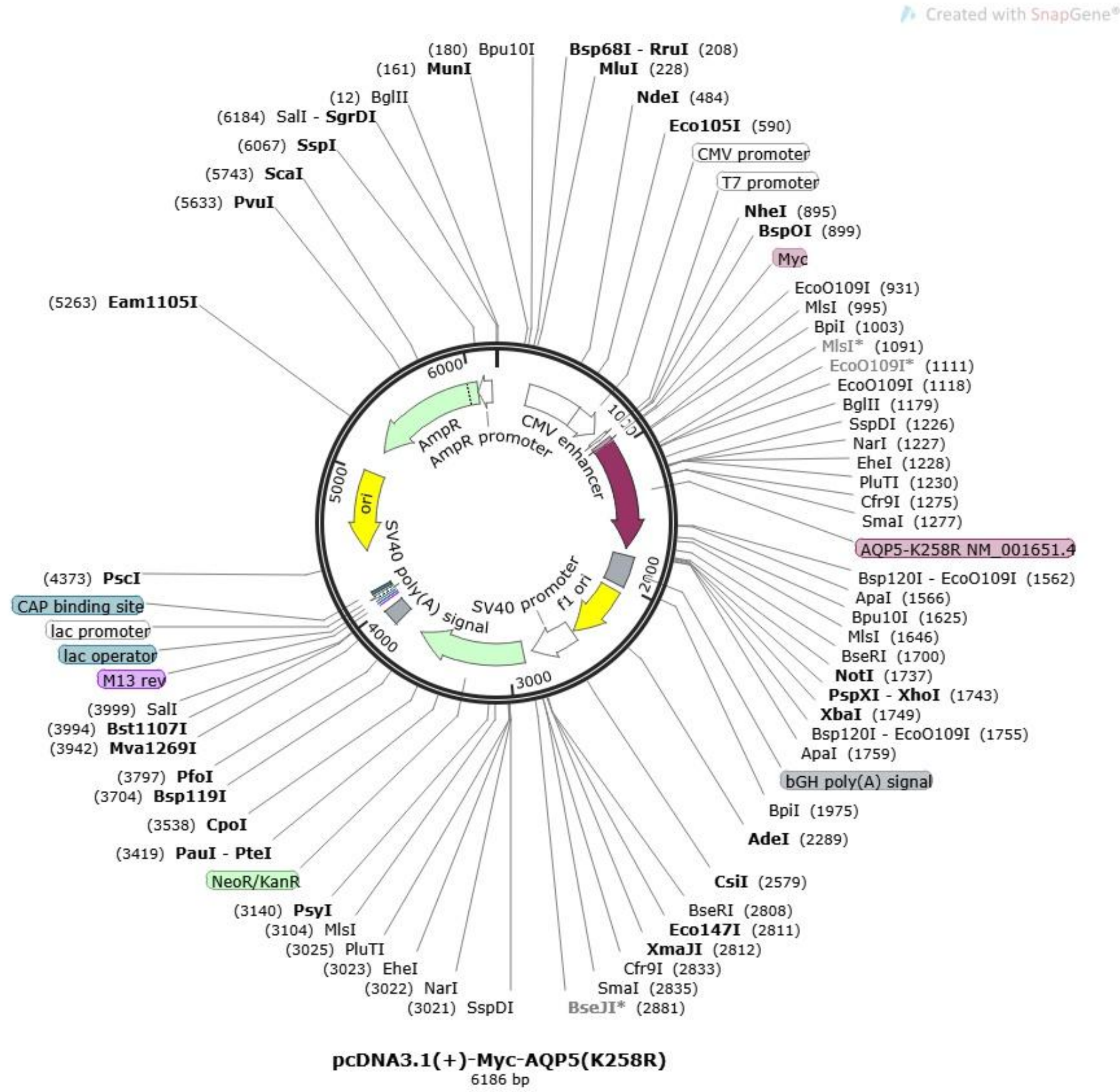

(E) Map of the pcDNA3.1-Myc-hAQP5(**K258R**) mutant vector (6186 bp). The construct encodes human AQP5 with a lysine-to-arginine mutation at position 258.

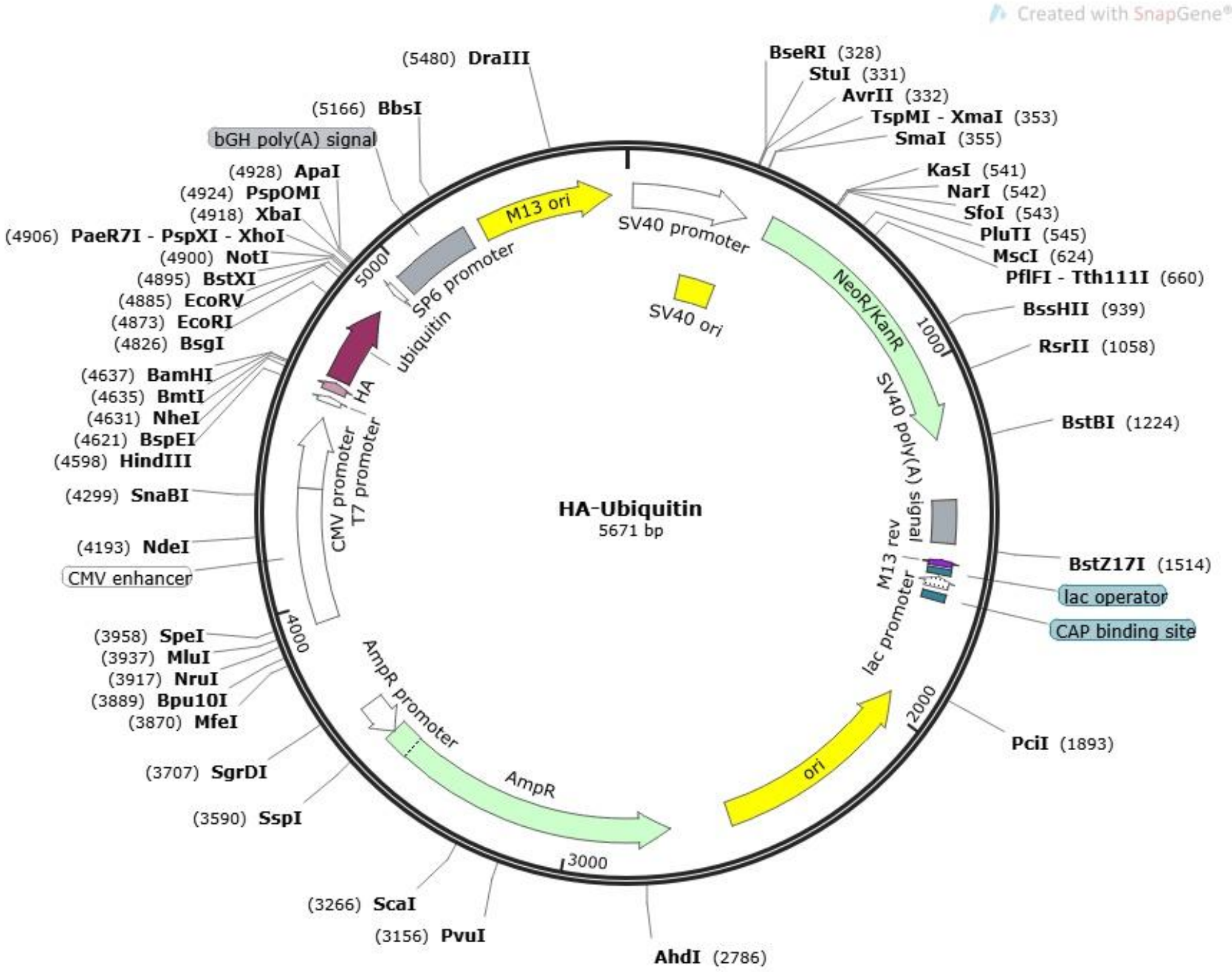

(F) Map of the **HA-Ubiquitin** vector (5671 bp). The construct encodes full-length human ubiquitin with an N-terminal HA tag.

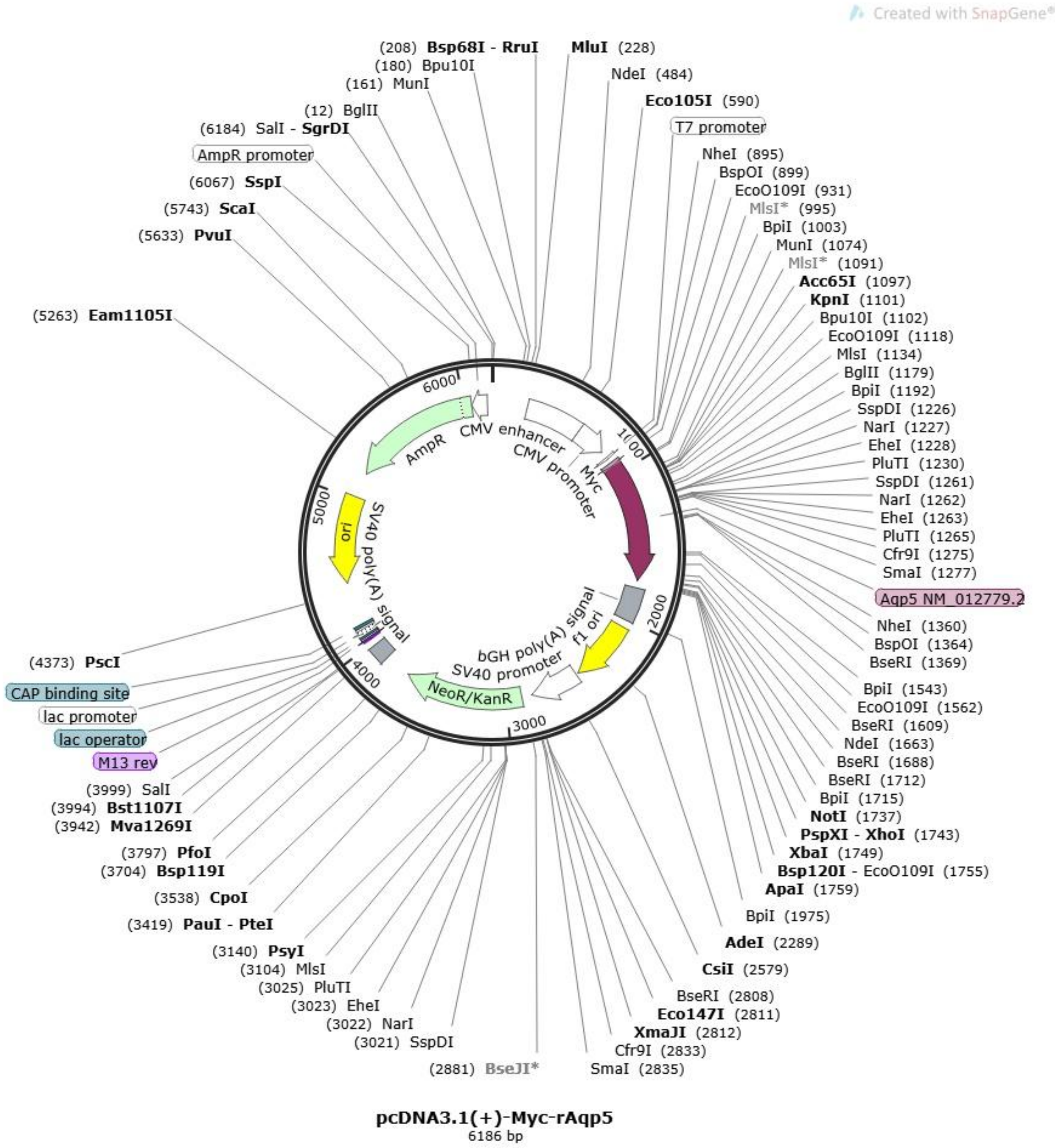

(G) Map of the pcDNA3.1-Myc-**rAqp5** vector (6186 bp). The construct encodes full-length rat AQP5 with an N-terminal Myc tag, used for in vivo overexpression experiments.

All constructs are expressed under the control of the CMV promoter/enhancer. The SV40 promoter drives the neomycin/kanamycin resistance gene for selection in mammalian cells, while the ampicillin resistance gene (Amp^r) is used for bacterial selection. Other key genetic elements, including the SV40 poly(A) and bGH poly(A) signals, are annotated in the maps. Plasmid maps were generated using SnapGene software. The complete annotated plasmid sequences are provided as SnapGene files in the Supplementary Materials.
