# Supplementary material for: The role of NEDD4-mediated ubiquitination of AQP5 in allergic rhinitis: implications for nasal mucosal barrier function
Source: Front Immunol. 2026 Sep 17;17:1908261. doi: 10.3389/fimmu.2026.1908261 (PMC13626906; doi:10.3389/fimmu.2026.1908261)
Supplement: Supplementary file 2 [file DataSheet2.pdf]

Supplementary Figure S2. Schematic diagram of the in vivo experimental design and treatment protocol.

Group	Systematic sensitization	Challenge	Intranasal Intervention
**NC**	Intraperitoneal injection (Saline)<br>Day 1 7 14	Intranasal instillation (Saline)<br>15 —— 21	Intranasal instillation (Saline)<br>22 25 28
**AR**	Intraperitoneal injection (OVA + $Al(OH)_3$)<br>Day 1 7 14	Intranasal instillation (OVA, 10 mg/mL)<br>15 —— 21	Intranasal instillation (Saline)<br>22 25 28
**AR + siNEDD4 (NEDD4 KD)**	Intraperitoneal injection (OVA + $Al(OH)_3$)<br>Day 1 7 14	Intranasal instillation (OVA, 10 mg/mL)<br>15 —— 21	Intranasal instillation (siNEDD4 liposome, 50 μg/rat)<br>22 25 28
**AR + ovAQP5 (AQP5 OE)**	Intraperitoneal injection (OVA + $Al(OH)_3$)<br>Day 1 7 14	Intranasal instillation (OVA, 10 mg/mL)<br>15 —— 21	Intranasal instillation (ovAQP5 liposome, 50 μg/rat)<br>22 25 28

SD rats were sensitized via intraperitoneal injection of OVA + $Al(OH)_3$ on days 1, 7, and 14. From day 15 to day 21, rats received daily intranasal OVA challenge to establish the AR model. On day 22, 25, and 28, rats in the treatment groups received intranasal administration of siNEDD4 or ovAQP5 plasmid (50 μg/rat), while control and AR model groups received saline. Nasal symptom scores were recorded daily during the final 3 days of OVA challenge (days 19–21). On day 29, rats received the final intranasal administration and were subsequently sacrificed for sample collection, including NLF for inflammatory cell counting, and nasal mucosal tissues for Western blot, qPCR, and histopathological (H&E) analysis. NC = normal control; AR = allergic rhinitis model; AR + siNEDD4 = AR model treated with siNEDD4; AR + ovAQP5 = AR model treated with ovAQP5 overexpression plasmid; OVA = ovalbumin; KD = knockdown; OE = overexpression.
